# Techniques for Local Anesthesia in Transperineal and Transrectal Prostate Biopsy: An Image-based Step-by-Step Guide With Dedication to Anatomy

**DOI:** 10.7759/cureus.80746

**Published:** 2025-03-17

**Authors:** Toni Franz, Theodoros Spinos, Julian Lüke, Tom Sicker, Benny Dinh, Hanno Steinke, Jens-Uwe Stolzenburg

**Affiliations:** 1 Department of Urology, University of Leipzig, Germany, Leipzig, DEU; 2 Department of Urology, University of Patras, Patras, GRC; 3 Department of Anatomy, University of Leipzig, Germany, Leipzig, DEU

**Keywords:** local anesthesia, pain management, prostate biopsy, prostatic plexus, transrectal ultrasound

## Abstract

Prostate biopsy is the cornerstone in the diagnostic pathway of prostate cancer. It’s normally performed using local or general anesthesia. The prostate's high sensitivity to mechanical stimuli and irritation is due to its complex nerve supply, mainly from the pelvic plexus. It originates from the sacral plexus, with both parasympathetic and sympathetic nerves having a role in prostatic innervation. As the procedure involves needle penetration into sensitive tissues, it is essential to ensure effective peribioptic pain management, as patients may experience considerable discomfort during the process. Local anesthesia has proven to be one of the safest, most reliable, and most effective approaches to managing this pain, as it provides targeted relief with minimal side effects. This report describes in detail the current anesthesia techniques applied during both transrectal and transperineal prostate biopsy procedures, delving into their mechanisms of action, as well as reviewing recent research findings on their efficacy. We describe an image-based step-by-step technique with dedication to pelvic anatomy for a comprehensive understanding of how local anesthesia can be administered and therefore enhance the overall experience of patients undergoing prostate biopsy.

## Introduction

Prostate biopsy is a crucial diagnostic procedure for prostate cancer [1,2]. The pain associated with the biopsy can be particularly intense in areas such as the prostate and surrounding structures, making pain prevention prior to invasive actions a critical aspect of patient care [3,4]. Local anesthesia is recognized as a safe, reliable, and effective method for managing this pain, offering targeted relief with minimal side effects [5]. 

While transrectal prostate biopsy is widely used, perineal biopsy has recently gained importance because it is often considered to be less prone to infection. The risk of infection and the necessity of antibiotic prophylaxis in a transperineal prostate biopsy, as one of several components of infection prevention, is a frequent topic of discussion, as recent studies suggest this technique allows for the avoidance of preoperative and peri-interventional antibiotic administration [6]. This could help reduce the risk of antibiotic resistance and side effects, which is especially important in times of rising antibiotic resistance. Nevertheless, performing the procedure can cause significant pain. Effective pain prevention is therefore essential to make the procedure more tolerable for the patient [5]. 

Local anesthesia is an efficient way to control this pain and has increasingly become the preferred method. Various techniques for local anesthesia have been developed and refined over the years to improve patient comfort and reduce the invasiveness of the procedure [7-17]. This article provides a detailed technical report on the current anesthesia techniques used for both transrectal and transperineal prostate biopsy procedures, based on a detailed comprehensive description of the anatomy of the nerves around the prostate. By explaining the different techniques, this report aims to provide a deeper understanding of methods for administering local anesthesia to optimize patient comfort during prostate biopsy.

## Technical report

Anatomy and pain mechanism

**Figure 1:**
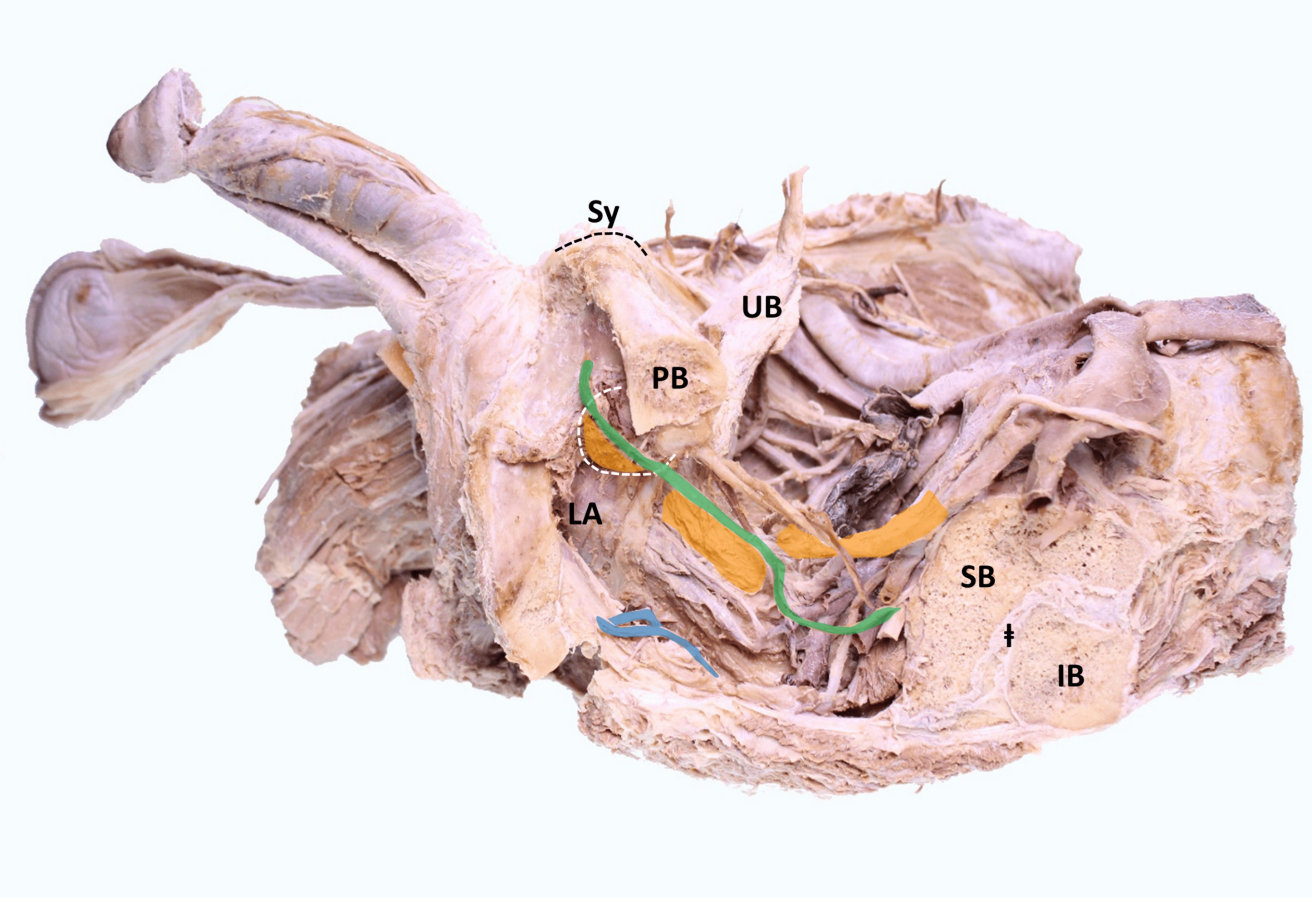
Preparation of a male pelvis in sagittal section Orange area: extension of the fibers of the pelvic plexus; Blue area: coccygeal plexus; Green area: pudendal nerve UB: urinary bladder; PB: pubic bone; LA: Levator ani muscle; SB: sacral bone; IB: ilium bone; ⱡ: sacroiliac joint. Image Credit: Hanno Steinke, author

**Figure 2:**
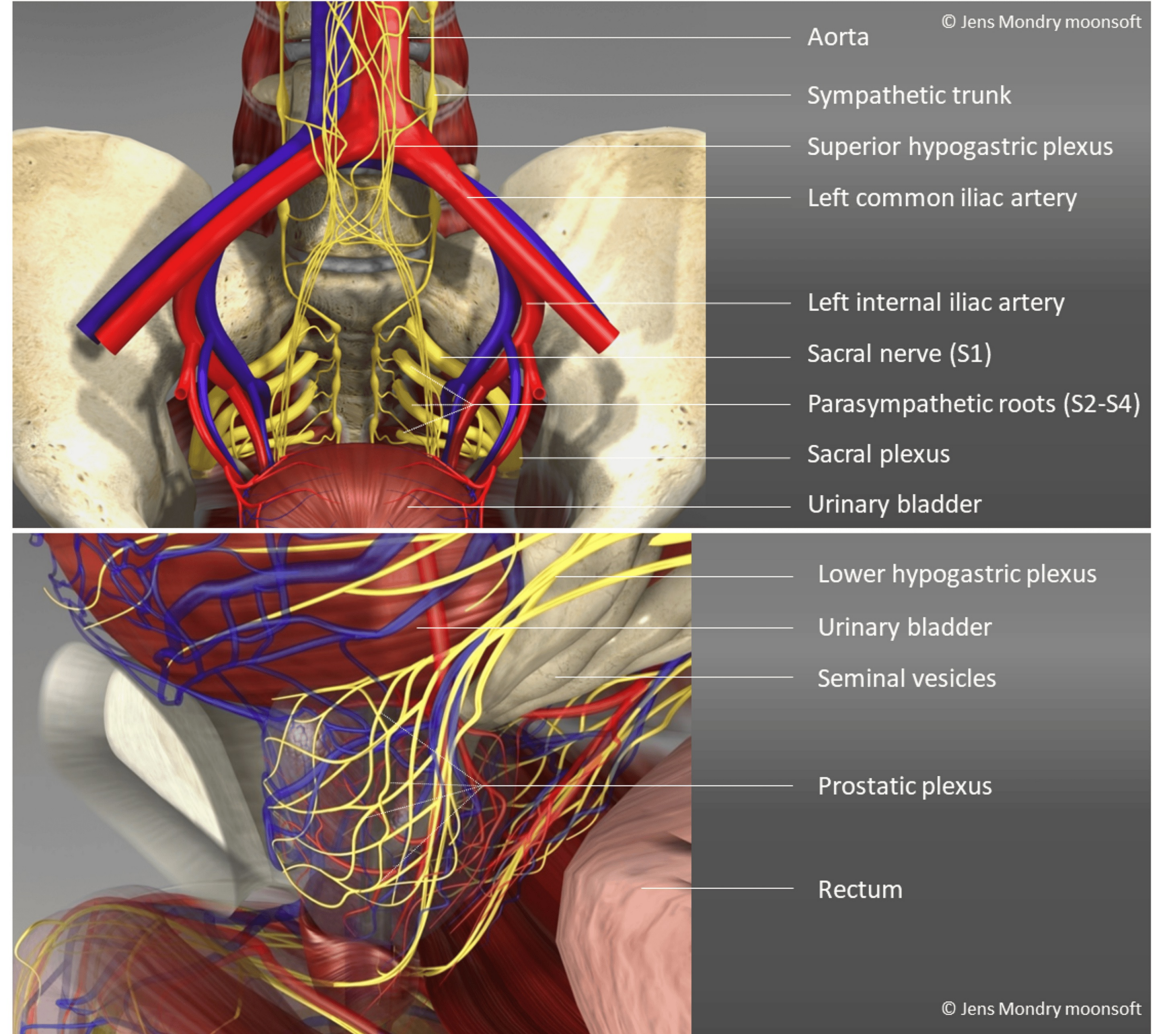
Nerve supply in the pelvis containing sympathetic and parasympathetic fibres (computer 3D visualization) Image Credit: Jens Mondry (Moonsoft, Jena, Germany); used with permission

The perineal approach to the prostate requires puncturing the skin, subcutaneous tissue, and muscles, all of which contribute to pain during the procedure. This is primarily caused by mechanical stimulation of nerve structures, particularly the pudendal nerve [18]. The prostate is sensitively supplied by a complex network of nerves and blood vessels. The neural supply is mainly provided by the pelvic plexus, a complex nerve network located in the pelvic area that contains both sympathetic and parasympathetic nerve fibers (Figures 1, 2) [19]. 

The high sensitivity of the prostate makes it an important component in the perception of pain in the pelvis. The nerves run from the lower parts of the spine (sacral region) and from the pelvic plexus. The prostate is innervated by these nerve fibers in terms of pain sensations, as well as in controlling ejaculation and bladder function. The pelvic plexus arises from the nerve roots S2 to S4 of the sacral spinal cord, also known as the sacral nerve plexus (Figure 2). These nerve roots form the inferior hypogastric plexus (lower hypogastric plexus), which extends into the pelvic plexus area. 

The pelvic plexus runs bilaterally along the pelvic wall, toward the prostate, and forms several branches [18,19]. The plexus contains both sympathetic and parasympathetic nerve fibers. Sympathetic fibers primarily originate from the lumbar and sacral parts of the sympathetic nervous system and are responsible for the contraction of prostate muscles and ejaculation. Parasympathetic fibers originate from the sacral segments S2 to S4 and are crucial for the relaxation of the prostate and the control of bladder functions. 

Branching and Innervation of the Prostate

The pelvic plexus gives off the prostatic plexus, which directly supplies the prostate. The prostatic plexus runs around the prostate, forming a dense network that contains both sensory nerves (responsible for pain sensations) and motor nerves (that control the muscle functions of the prostate and the regulation of ejaculation) (Figure 2) [19]. 

Connection to the Vesical Plexus

A portion of the parasympathetic fibers from the pelvic plexus continues to the vesical plexus, which innervates the bladder, enabling a close functional connection between the prostate and bladder in the control of urination and ejaculation. The sympathetic part of the plexus is responsible for the contraction of prostate glands and plays a role in ejaculation. The parasympathetic part is responsible for the blood supply to the prostate and its relaxation, which is also significant for sexual function and the control of bladder function (Figures 1, 2) [19-23].

Local anesthesia techniques

Several local anesthesia techniques are applied to prevent pain during perineal prostate biopsy including the modalities given below.

Infiltration Anesthesia

This technique, where the anesthetic is directly injected into the skin and tissue of the perineum, is most commonly used. It has been shown to be effective in reducing pain at the injection site [7,8,9]. Lidocaine and bupivacaine are the generally applied anesthetics, with lidocaine providing rapid onset and relatively short-lasting effects, while bupivacaine offers longer-lasting pain relief [24,25].

Pudendal Nerve Block

A targeted block of the pudendal nerve is another possible method to alleviate pain in the pelvic region. It is performed in the lithotomy position, with the procedure guided by digital rectal examination. The anesthetic is injected percutaneously just behind the ischial spine, near the sacrospinous ligament, without the aid of electrostimulation or radiologic imaging. The needle is directed at a 45° angle into each ischiorectal fossa, with about 10 ml of the anesthetic solution injected into both sides, using aspiration to avoid vein entry. This approach blocks the inferior rectal nerve and the posterior pudendal nerve. The needle can then optionally be advanced further until it reaches the presacral fascia, where 5 ml of anesthetic are injected to block the anococcygeal nerves. A limitation of this method may be that it requires highly precise anatomical knowledge. Complications are rare. The most common adverse event is discomfort at the injection site. The risk of bleeding and infections is less frequent. More serious adverse events occur rarely and include damage to the nerve itself or injury to adjacent organs such as the bladder and rectum. Puncture of the pudendal artery with intravascular injection of local anesthetics may lead to systemic local anesthetic toxicity, which can be potentially fatal [10,26,27].

Periprostatic Anesthesia

Periprostatic anesthesia, where the anesthetic is injected directly into the tissue around the prostate, is a highly effective method for blocking pain receptors in the prostate itself [3,11-17,28]. A randomized controlled study by Berger et al. showed that this technique offers significant pain relief and improves patient satisfaction compared to placebo [29]. Also Wang and colleagues considered the blockade of the branches of the pelvic plexus to be safe, effective, and repeatable for local anesthesia in transperineal prostate biopsies [9].

Intrarectal Anesthesia

Another technique for pain reduction during prostate biopsy is intrarectal administration of anesthetics or analgetics such as lidocaine or diclofenac [7,30]. Chang et al. [31] and Leung et al. [32] conducted studies that showed that intrarectal lidocaine gels significantly reduce pain during biopsy, although their effect may not always be comparable to other methods, such as periprostatic anesthesia.

Alternative Anesthesia Methods

The use of oral analgesics, such as selective COX-2 inhibitors, was also investigated, without showing a significant pain-relieving effect. Moinzadeh found that oral rofecoxib prior to biopsy did not significantly reduce the severity of discomfort in patients [13]. Although local anesthesia is often sufficient, in some cases, the expertise of anesthetists may be necessary. This is particularly worth considering for patients with high pain sensitivity or for more complex procedures, where intravenous sedation, epidural anesthesia, or even intubation anesthesia may be considered. However, these methods are associated with a higher risk of side effects and require longer post-procedure monitoring [33]. In a group of 30 patients, Nippon et al. demonstrated that transurethral incision of the prostate (TUIP) under local anesthesia is well tolerated and recommended for high-risk patients with small benign prostatic hyperplasia [34].

Efficacy, Safety, Advantages, and Disadvantages of Local Anesthesia

The efficacy of local anesthesia during perineal prostate biopsy has been evaluated in numerous studies. It has been shown that infiltration with lidocaine or bupivacaine provides effective pain relief, allowing patients to resume their normal activities quickly after the procedure [17]. However, a significant challenge remains that some patients may not experience complete pain relief, necessitating additional injections or a combination with other anesthesia methods. The safety of local anesthesia is generally high. Several studies indicate that severe complications such as allergies or systemic reactions are rare, especially when the anesthetic doses are administered correctly [35]. However, possible side effects such as hypotension or numbness at the injection site should be taken into account.

Local anesthesia offers several advantages and disadvantages that are important to consider in clinical practice [36-40]. One of its key benefits is the minimal systemic impact it has compared to other forms of anesthesia such as general anesthesia. Additionally, it is characterized by a rapid onset and is relatively simple to administer, making it a practical choice for many procedures. Local anesthesia also minimizes postoperative recovery time and is generally well-accepted by patients due to its less invasive nature compared to alternative methods [36,37,40]. However, local anesthesia is not without its limitations. In some cases, particularly with deeper biopsies, it may not provide sufficient pain relief. Achieving the desired effectiveness often requires precise technique, which may pose a challenge in certain situations. Furthermore, there is a risk of local side effects such as hematomas or infections at the injection site, which must be carefully managed to ensure patient safety [36,37,40]. A severe, potentially life-threatening complication associated with local anesthesia is Local Anesthetic Systemic Toxicity (LAST). This condition can occur when an excessive dose of a local anesthetic enters the systemic circulation. In a review article by Neal, which examined various types of local anesthesia, the incidence of LAST was reported as 0.59 per 1000 procedures under ultrasound guidance and 2.1 per 1000 procedures without ultrasound guidance [37]. The timing of onset, the severity of initial manifestations, progression to cardiovascular impairment, as well as the duration and recurrence of toxicity, reflect the interaction of a large number of independent variables, including the choice of local anesthetic, the anesthetic dose, the injection rate, the site of injection, the patient's comorbidities, and the individual patient's sensitivity to the local anesthetic [39]. A study involving more than 100,000 local/regional anesthesia documented complications clearly attributable to the type of anesthesia [38]. Among these, more than 21,000 were regional anesthesias. Overall, complications were rare, the majority of which were mild (pain or redness at the injection site). Severe complications occurred in a total of 98 cases. In the context of regional anesthesias, three cardiac arrests occurred. Of 34 neurological complications (radiculopathy, cauda equina syndrome, paraplegia), 21 were associated with either paresthesia during puncture (n = 19) or pain during injection (n = 2), suggesting nerve trauma or intraneural injections [38].

Local anesthesia in transrectal access

**Figure 3:**
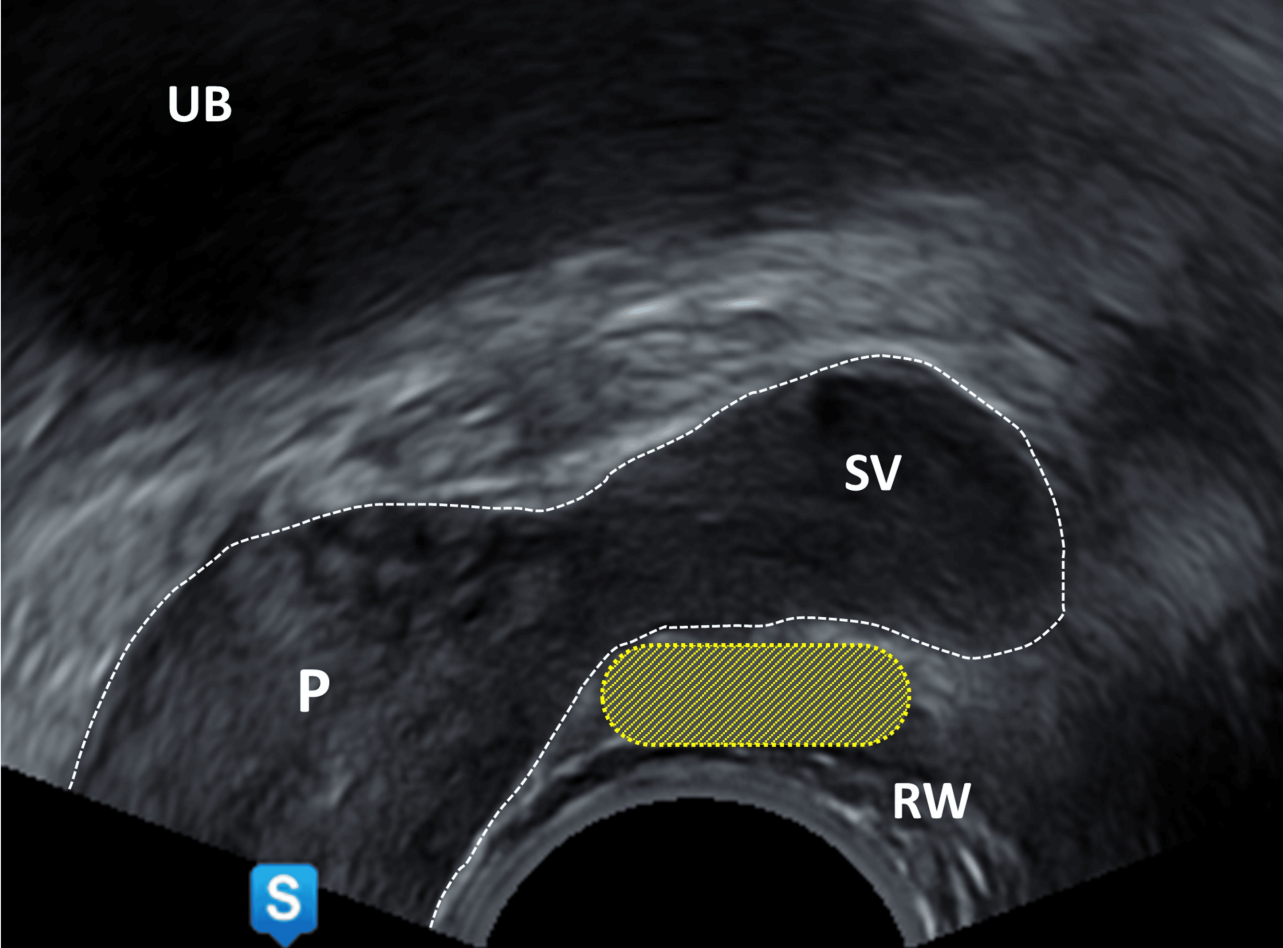
Sagittal section of the prostate, ultrasound probe slightly rotated Yellow area: area of interest for local anesthesia  between SV, P, and RW UB: urinary bladder, P: prostate, SV: seminal vesicle, RW: rectal wall Image Credit: Toni Franz, corresponding author

The patient is positioned either in the lithotomy position or left lateral position, depending on the surgeon's preference and the spatial conditions. Since the patient will remain in this position for about 15 minutes, it contributes to the patient's comfort if they lie comfortably. First, a sterile lubricating gel with a local anesthetic (e.g., 2% lidocaine gel) is applied into the anus to achieve surface anesthesia of the intestinal mucosa, making pain caused by the subsequent periprostatic local anesthesia more tolerable. During digital rectal examination, the gel is spread and massaged into the mucosa. Next, the transrectal ultrasound probe is inserted, the prostate is measured, and its zonal structure, parenchymal composition, and capsule boundary are assessed. Later, the local anesthesia is applied; ideally performed in the sagittal plane. Therefore, we use a 22G x 220 mm needle (Chiba; Pajunk GmbH, Geisingen, Germany). A total of 10 ml of 1% xylocaine is sufficient. Special attention is given to the area between the prostate base, the seminal vesicles and rectum. For this, the probe must be rotated slightly laterally from the midline (Figure 3). 

**Figure 4:**
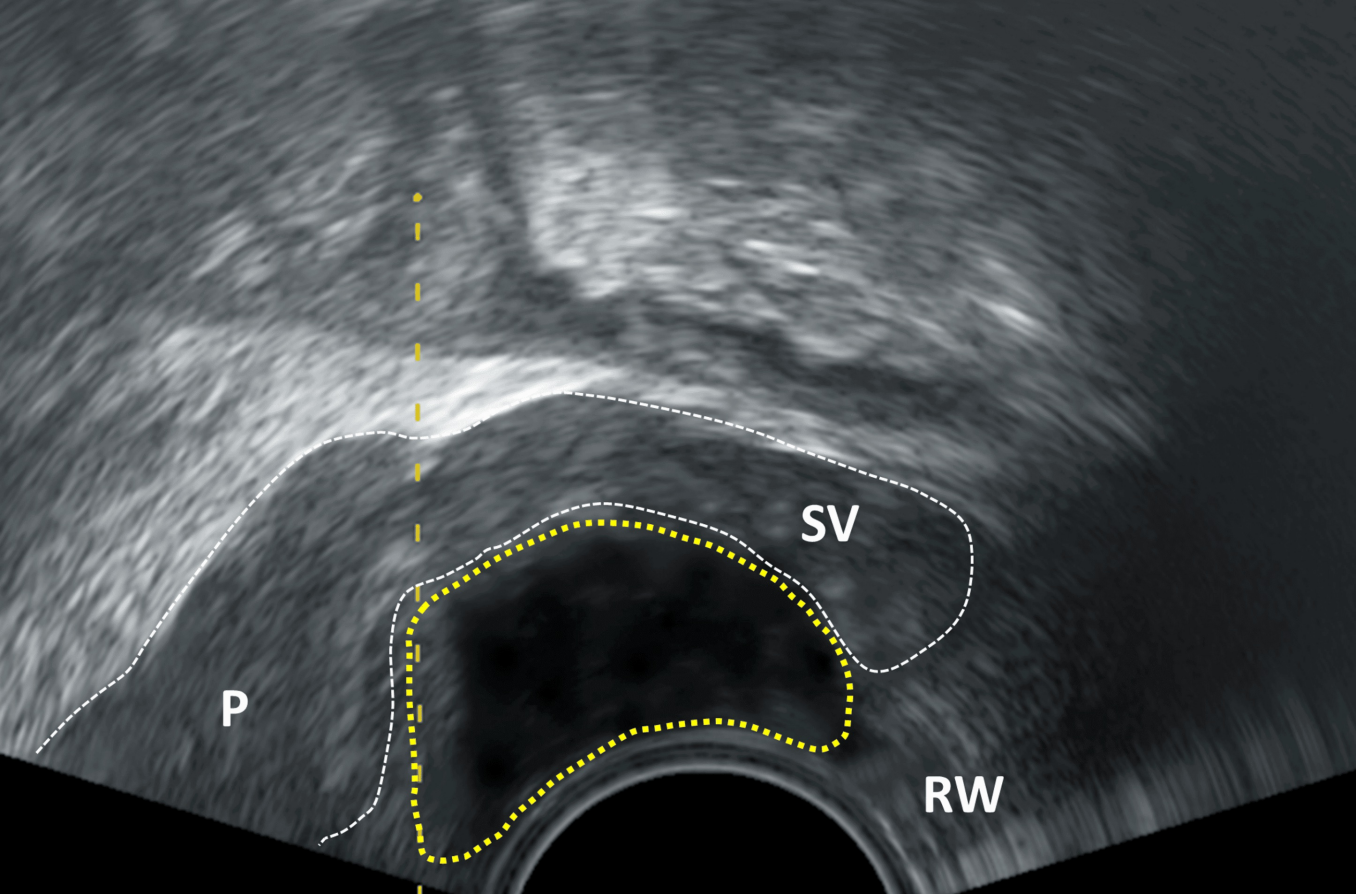
Sagittal section of the prostate P: prostate; SV: seminal vesicle; RW: rectal wall; area of yellow dotted line: applicated local anesthetic as echofree cushion which elevates the seminal vesicle; yellow dashed line: needle track Image Credit: Toni Franz, corresponding author

**Figure 5:**
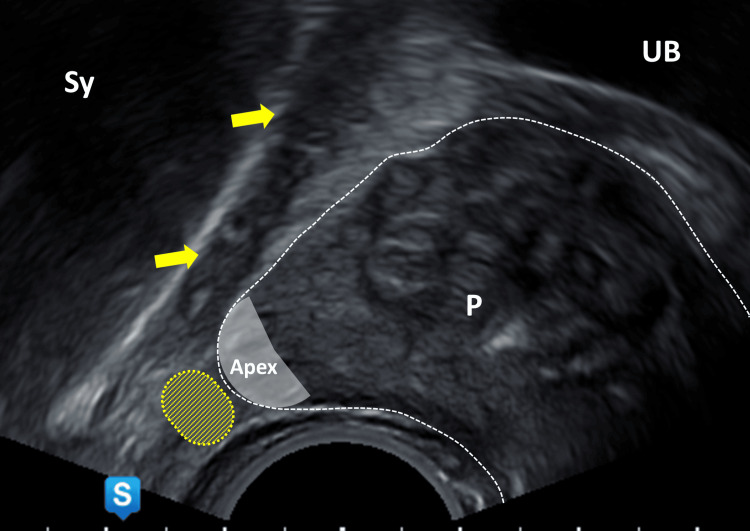
Sagittal section of the prostate, midline, focus on the apex UB: urinary bladder; P: prostate; Sy: symphysis; grey area: prostatic apex; yellow area: area of interest for local anesthesia; yellow arrows: muscular pelvic floor Image Credit: Toni Franz, corresponding author

**Figure 6:**
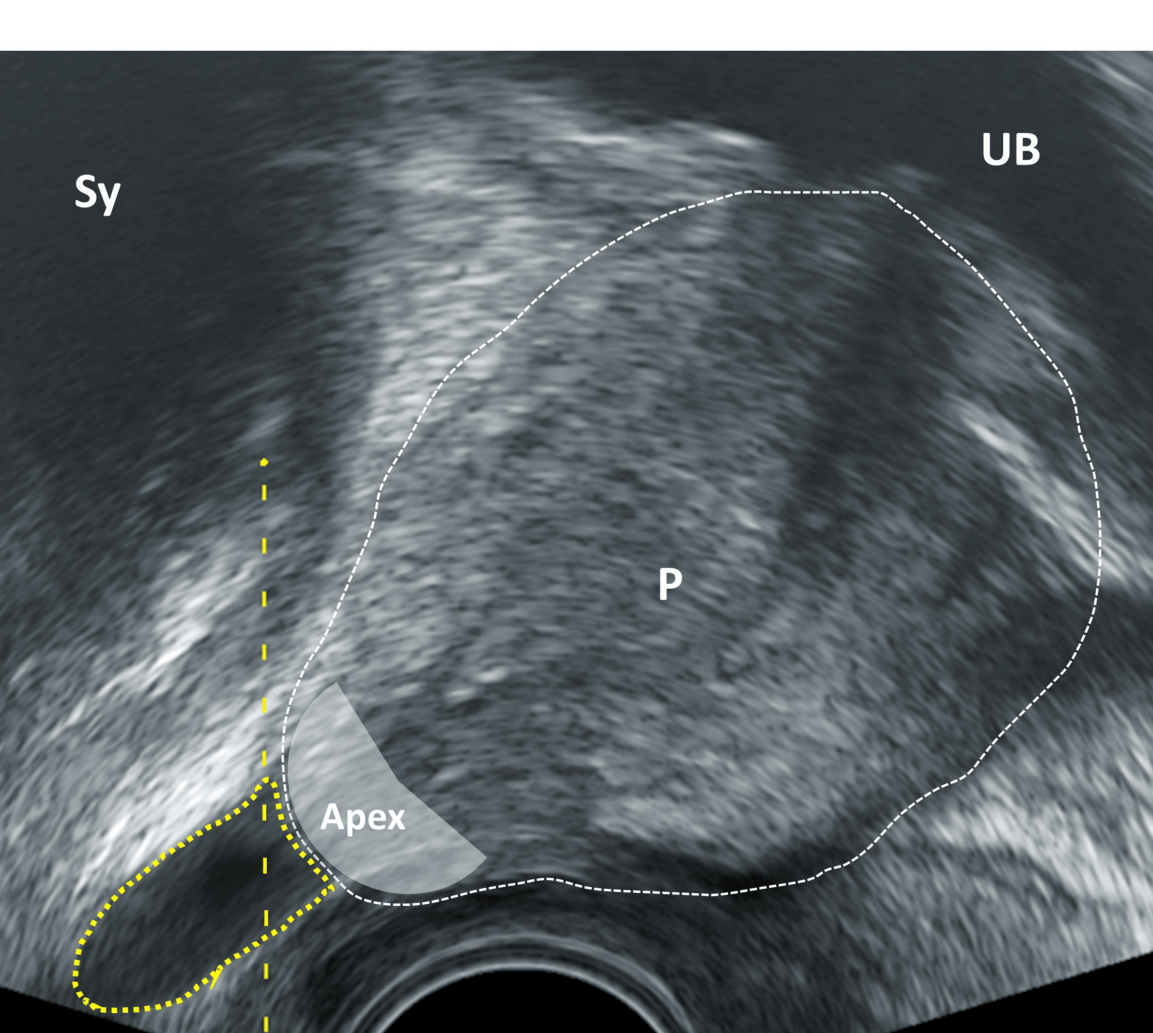
Sagittal section of the prostate, focus on the apex UB: urinary bladder; P: prostate; Sy: symphysis; grey area: apex; area enclosed by yellow dotted circle: echofree cushion after injection of local anesthetic; yellow dashed line: needle track Image Credit: Toni Franz, corresponding author

At this point, 4 ml is injected on each side into the narrow space between the rectum and the seminal vesicle. If the needle tip is in the correct position, the seminal vesicle is lifted by the injected volume and thereafter appears like sitting on a cushion (Figure 4). If this does not occur, the position of the needle tip should be checked to ensure it has not been inserted too far (injection into the seminal vesicle) or too shallow (injection into the rectal wall or perirectal fat). The needle tip itself appears on ultrasound as a distinct, highly echogenic reflection with posterior sound shadowing and can therefore be easily identified. The same procedure is now carried out on the opposite side. After sufficient application at the prostate base, the focus is directed towards the prostatic apex. The remaining 2 ml is applied directly near the capsule (precapsular) along the midline (Figures 5, 6). Care must be taken not to perforate the urethra or the capsule itself.

Local anesthesia in transperineal access

**Figure 7:**
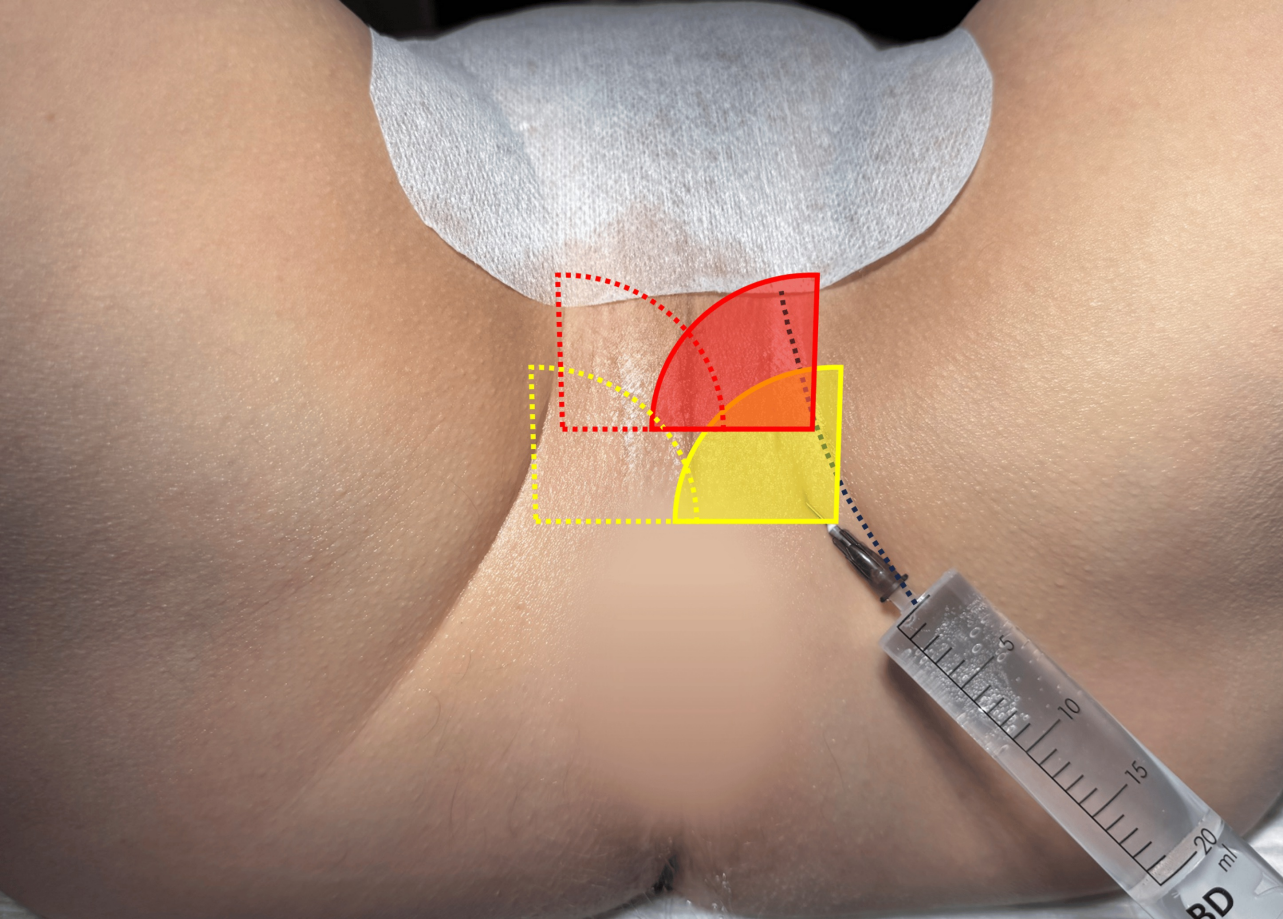
Prepared perineum in lithotomy position, scrotum fixed by tape Blue dotted line: banana-shaped skinfold; yellow area: first superficial anesthesia, area of effect of the local anesthesia; red area: second superficial anesthesia; yellow plus red: overlapping areas of effect with complete superficial anesthesia of the perineum Image Credit: Toni Franz, corresponding author

The patient is positioned in the lithotomy position. The scrotum is displaced toward the penis/mons pubis, either manually by the patient himself or with the use of adhesive tape. The patient holding it himself offers two advantages: firstly, the patient is engaged and thus distracted, and secondly, the pain caused by removing the tape is avoided. Depending on the patient's constitution, it may be useful to tilt the operating table by about 5-10° into the Trendelenburg position. This can help facilitate access to the perineum. Next, the perineum is shaved and disinfected sterilely (Figure 7).

Initially, surface anesthesia of the perineum is performed. Therefore, a superficial fan shaped skin anesthesia is created with approximately 3 ml of 1% xylocaine injected into the banana-shaped skin fold between the perineum and the inner thigh, with the needle directed from laterocaudal to mediocranial (Figure 7). In this process, the formation of a small wheal is desirable. Therefore, we use a 22G x 30 mm injection needle (Sterican; B. Braun Melsungen SE, Melsungen, Germany). The previously anesthesized skin area is then punctured, and another subcutaneous depot with approximately 3 ml is created in the same direction from laterocaudal to mediocranial. 

**Figure 8:**
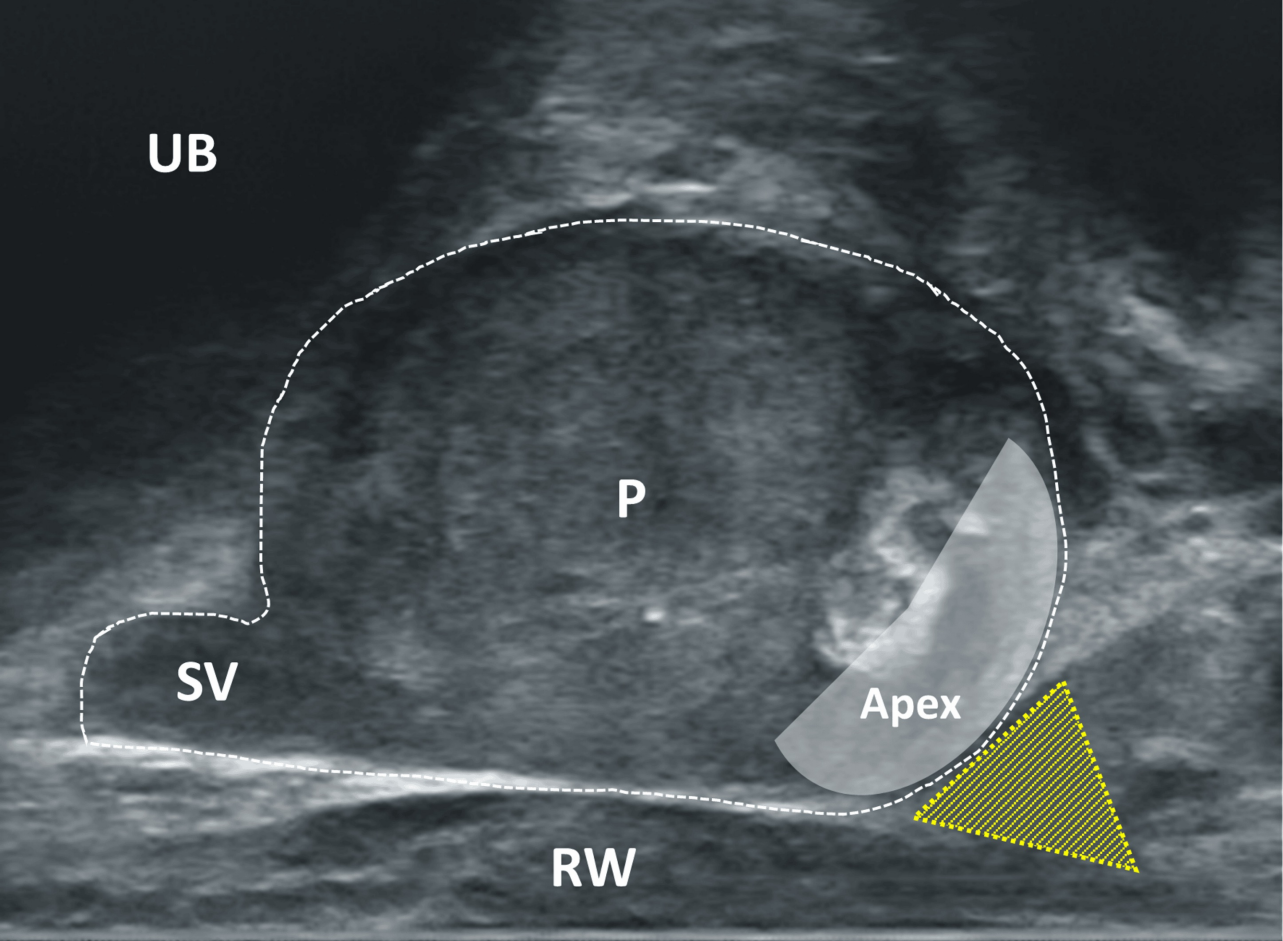
Sagittal section of the prostate, ultrasound probe medially UB: urinary bladder; P: prostate; SV: seminal vesicle; RW: rectal wall; yellow area: area of interest for local anesthesia - preapical triangle between P, RW, and pelvic floor; grey area: apex Image Credit: Toni Franz, corresponding author

**Figure 9:**
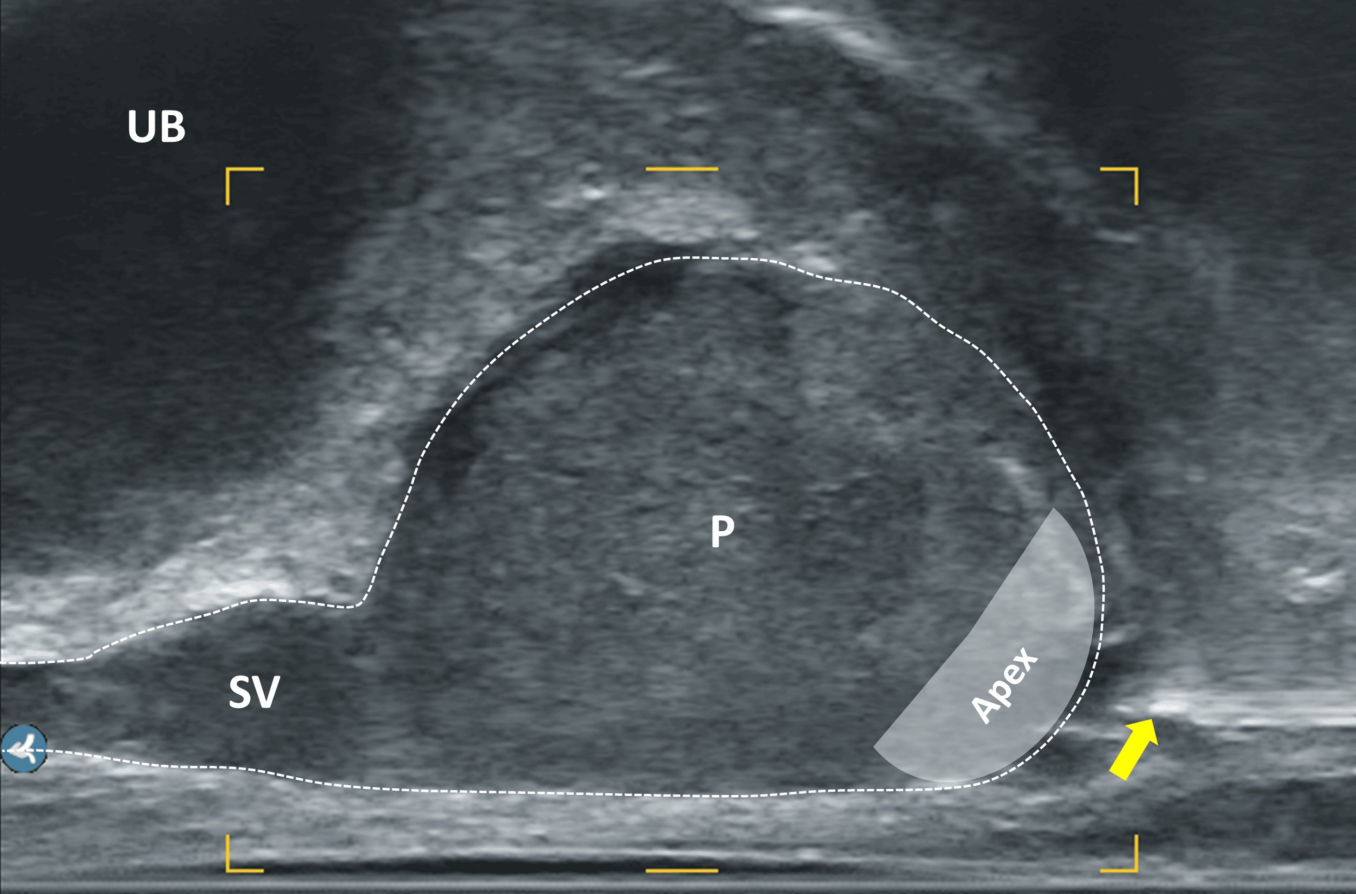
Sagittal section of the prostate, ultrasound probe medially UB: urinary bladder; P: prostate; SV: seminal vesicle; yellow arrow: needle tip in the area of interest in the preapical triangle with local anaesthetic; grey area: apex Image Credit: Toni Franz, corresponding author

It is important to cross the midline (raphe perinei) during the process to achieve a complete effect (Figures 8, 9). The procedure is repeated on the opposite side, ensuring that the anesthetized areas overlap and the entire perineum is superficially anesthetized (Figure 7). The transrectal probe is then inserted, the prostate measured, and its zonal structure, parenchymal composition and capsule boundaries are assessed. The periprostatic local anesthesia is performed in the sagittal plane, with needle insertion via the puncture canal of the ultrasound probe. Using transrectal ultrasound guidance, local anesthetic is administered along the needle path on both sides around the puborectal muscle. The needle is inserted up to the precapsular region at the apex, where the prostate and rectum form a small triangle (Figure 8). The remaining 8 ml ( 12 ml used in the steps before) of 1% xylocaine are now used, with the probe being slightly rotated laterally, and approximately 4 ml per side are injected paramedian. 

**Figure 10:**
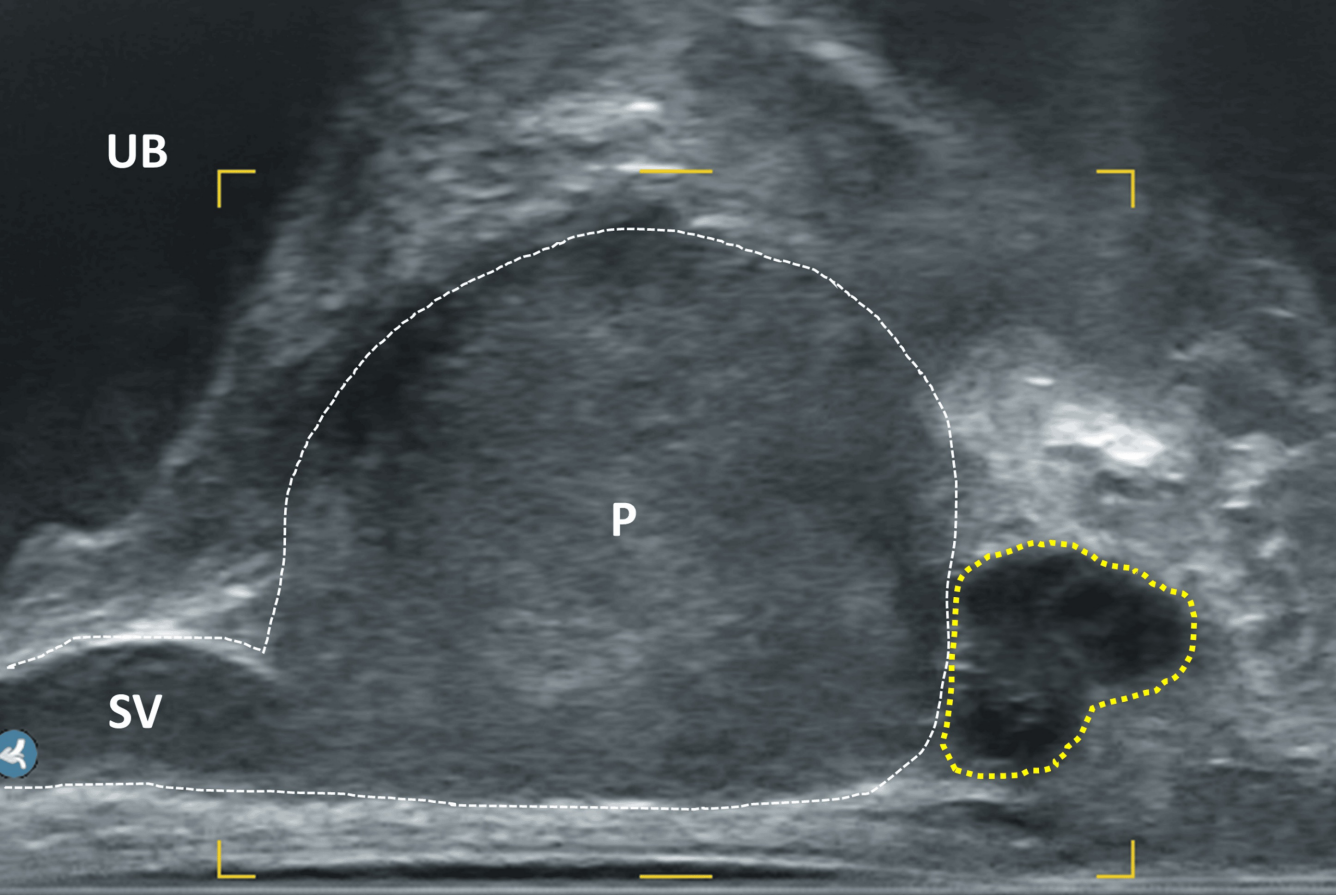
Sagittal section of the prostate UB: urinary bladder; P: prostate; SV: seminal vesicle; yellow dotted area: local anesthetic as echofree cushion at the prostatic apex Image Credit: Toni Franz, corresponding author

**Figure 11:**
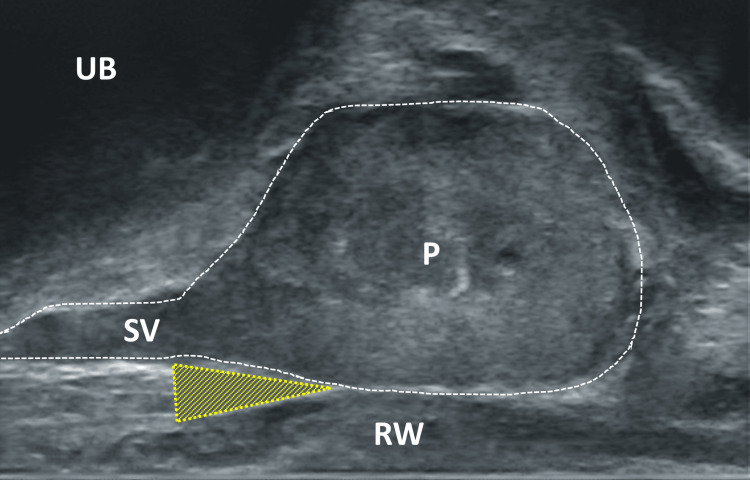
Sagittal section of the prostate, ultrasound probe slightly rotated UB urinary bladder; P prostate; SV seminal vesicle; RW rectal wall; yellow triangle: area of interest for local anesthesia between P, RW, and SV Image Credit: Toni Franz, corresponding author

**Figure 12:**
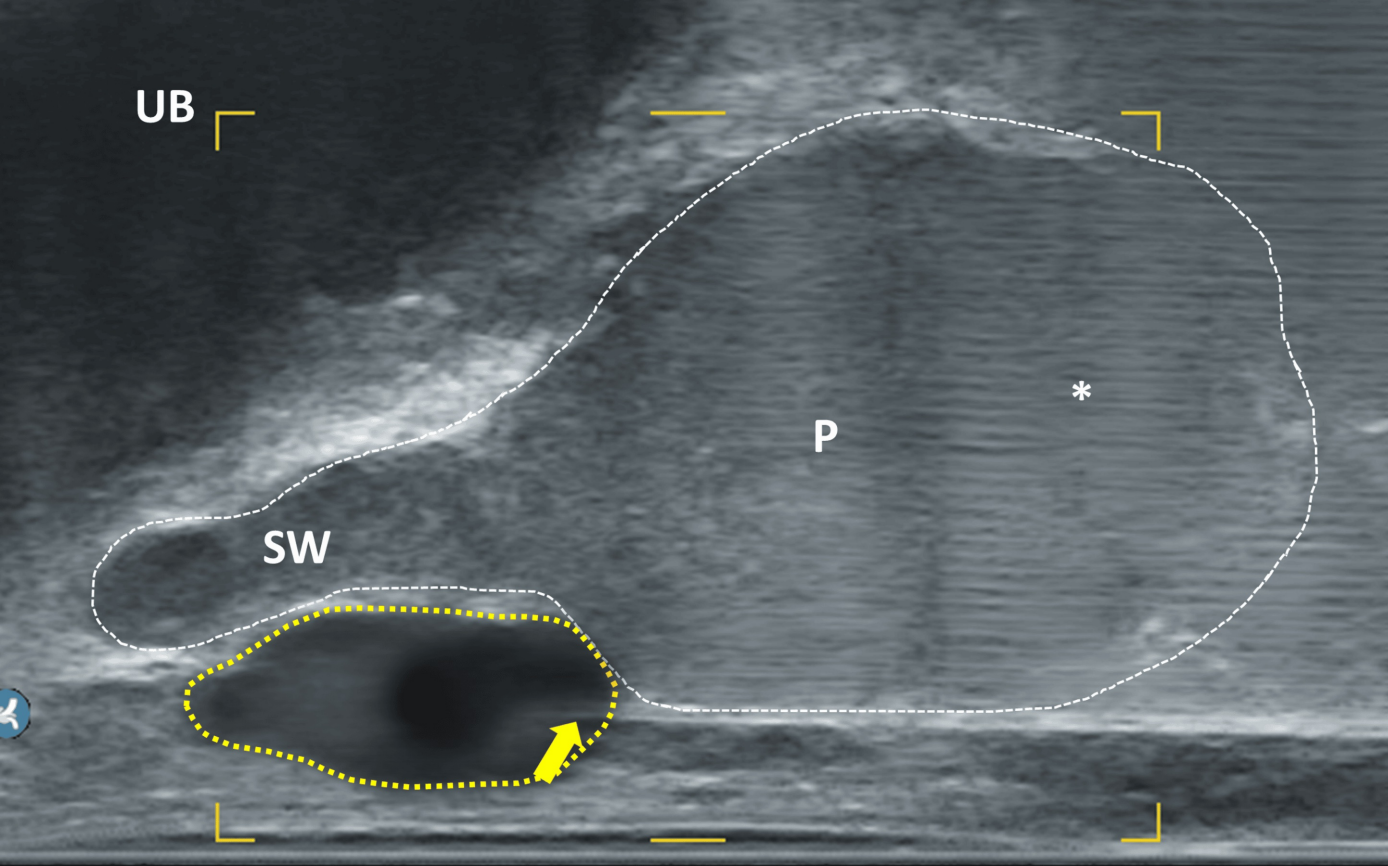
Sagittal section of the prostate UB: urinary bladder; P: prostate; SV: seminal vesicle; area enclosed by yellow dotted line: local anesthetic as echofree cushion below the seminal vesicle; yellow arrow: needle tip; *artifact due to the ultrasound extinction by the needle Image Credit: Toni Franz, corresponding author

If the needle tip is in the correct position, the local anesthetic should appear as an echoless area within the triangle (Figures 9, 10). If this is not the case, the position of the needle tip should be checked to ensure it has not been inserted too far (which would result in an injection into the prostatic apex) or too shallow (which would result in an injection into the pelvic floor muscles). The needle appears as a distinctly echogenic double contour with a posterior acoustic shadow and can thus be easily verified. As a countercheck to ensure that the needle is directly at the apex of the prostate, it is possible to gently advance the needle repetitively to tap and "palpate" the capsule. This helps prevent both under-insertion of the needle and over-insertion, which could perforate the capsule. Anesthesia in the area of the prostate base is optional but recommended for larger glands, a large number of planned biopsies or regions of interest in the basal parts of the gland. Therefore, the probe is rotated a bit laterally until the triangle between the prostate base, seminal vesicle and rectum is visible (Figure 11). The needle can then be carefully advanced between the backside of the prostate and the rectum wall toward the base, into the described triangle. In this area, 4 ml is injected on each side. It is crucial to rotate the probe to the opposite side only after the needle has been retracted into the needle guidance to avoid severe injury of the rectum. When the needle tip is in the correct position, the seminal vesicle should appear to be elevated like sitting on a cushion of the injected volume (Figure 12). If this does not occur, the position of the needle tip should be re-checked.

## Discussion

Transrectal and transperineal prostate biopsies are key methods for diagnosing prostate cancer [1,2]. The sextant biopsy, involving six systematically distributed prostate samples, has been the most widely utilized biopsy method over the past decade. Because the procedure was traditionally considered minimally painful, it was assumed that anesthesia was unnecessary [29]. However, a single set of sextant biopsies will fail to diagnose prostate cancer in up to 34% of men with the disease [14,41,42]. Because of this miss rate, many centers now obtain a larger number of biopsy specimens to improve cancer detection [29]. With 10 or more biopsies now being the standard, this perspective has shifted. Advancements in biopsy techniques like MRI/transrectal ultrasound (TRUS)-fusion, including the increased number of needle cores, have highlighted the need for effective pain management to enhance patient comfort. Given the procedure's potential to cause significant pain due to needle penetration of sensitive tissues, effective local anesthesia is crucial. Studies indicate that the pain intensity after surgery with the transperineal approach is significantly higher than with the transrectal method [4]. Since patients undergoing prostate biopsy are often elderly with low pain tolerance, higher pain levels may negatively affect the accuracy of the biopsy and the postoperative recovery process [6]. Studies have also demonstrated that pain intensity associated with the procedure increases with the number of puncture needles used [4,6,43]. About half of the patients who did not receive anesthesia experienced severe to intolerable pain during the prostate biopsy. Two other studies, conducted by Collins et al. [44] and Clements et al. [5], revealed that 65% and 90% of patients, respectively, reported significant discomfort and pain. Irani et al. reported that approximately 19% of patients would refuse to undergo a repeat biopsy without some form of anesthesia [45].

Although the transperineal approach has obvious advantages, its puncture path requires passing through the skin, fascia, and various layers of muscle tissue, which makes it more pain-sensitive. Over the past decade, numerous authors have discussed both the pain associated with the procedure and the effectiveness of various types of local anesthesia. Local anesthesia is an established and effective method for pain management during transrectal and transperineal prostate biopsy. It offers significant pain relief with lower overall risks compared to other anesthesia techniques and allows for quick recovery. Several techniques have been described to alleviate the pain associated with prostate biopsy, including spinal anesthesia, pudendal nerve block, peripheral prostate nerve block, and general anesthesia, all of which have been refined to ensure efficient pain management by targeting specific nerve structures while minimizing side effects [4,7,8,10-14,16,17,30,31,33,34,42,46,47]. For example, a study focusing on perineal nerve branches showed that injecting lidocaine between the deep layer of the superficial fascia and the prostate capsule under transrectal ultrasound guidance resulted in the most effective reduction of pain during transperineal biopsy [4]. Additionally, reports suggest that transperineal biopsy under local anesthesia is well tolerated by most patients. However, reports on the effectiveness of these methods have varied [15,48]. The effectiveness of specific anesthesia methods remains variable across studies. In a study conducted by Nash et al. in 1996, 64 patients scheduled for sextant prostate biopsies were evaluated in a placebo-controlled trial [47]. The treatment group received a nerve block with 5 ml of 1% lidocaine on one side of the prostate, while the contralateral side was injected with 0.9% saline. The results showed a significantly lower pain score on the anesthetized side compared to the non-anesthetized side. Soloway et al. confirmed in 2000 that a nerve block of the prostatic plexus reduces the pain associated with sextant biopsies and recommended its routine use [35]. However, Wu et al. demonstrated in a randomized, double-blind study that this method has no significant effect on pain during a 12-core biopsy [3]. 

Another approach, described by Costello et al., involves placing an anesthetic depot near the seminal vesicles [11]. This technique requires experience in interpreting TRUS images and access to high-resolution ultrasound devices. By contrast, apex anesthesia is easier to perform with standard TRUS probes. In this method, the anesthetic is administered along the dorsocaudal surface of the apex capsule, achieving an effective nerve block in a region with high nerve density. Berger et al. introduced a modified apex anesthesia technique using only 2 ml of 2% lidocaine per side, which also resulted in significant pain relief [29]. Similarly, Taverna et al. demonstrated in 2002 that injecting 10 ml of 1% lidocaine into the midline between Denonvilliers’ fascia and the prostate surface effectively reduces pain during prostate punctures [8]. This approach has the advantage of requiring fewer injections. 

The administration of oral non-steroidal anti-inflammatory drugs (NSAIDs) has also been investigated. Moinzadeh et al. found in a placebo-controlled study that administering rofecoxib, a selective cyclooxygenase-2 inhibitor, one to two hours before the biopsy did not significantly reduce pain [13]. In contrast, Haq et al. reported that rectal administration of 100 mg diclofenac suppositories significantly alleviates pain without increasing morbidity [33]. These findings were further supported by Bach et al., who demonstrated a 48.3% reduction in pain compared to the control group with 50 mg diclofenac suppositories administered 30 minutes prior to the biopsy [30]. The potential for further pain reduction by combining diclofenac suppositories with apex anesthesia remains an area for future research. Another method, transperineal pudendal nerve block, was studied by Adsan et al. in a placebo-controlled, double-blind trial involving 65 patients, showing significant pain relief [10]. However, this study did not account for the pain caused by the anesthesia procedure itself. Studies on the intrarectal application of lidocaine gel have yielded conflicting results. While Leung et al. found no significant pain reduction using 2% lidocaine gel prior to a 10-core biopsy [32], Raber et al. reported that applying 5 ml of 2% lidocaine gel before a 12-core biopsy resulted in significant pain relief [7]. Notably, pain intensity tends to increase over the course of the biopsy procedure, with later cores perceived as more painful. For patients with low pain tolerance or undergoing biopsies requiring a high number of needle punctures, general anesthesia should also be considered.

## Conclusions

The choice of the specific technique should be tailored to the patient's needs and the individual biopsy case, with pudendal nerve block and periprostatic anesthesia being promising options for comprehensive pain relief in most cases. For this, it is helpful to address the patient's individual medical history during the preoperative discussion and explicitly inquire about previous experiences with anesthesia, potential allergies, psychological factors, as well as general preferences regarding anesthesia. Additionally, age and physical condition should be considered in the decision-making process. The current state of scientific knowledge shows that local anesthesia during prostate biopsy significantly reduces patients' pain perception without compromising the procedure. This method enhances patient satisfaction and provides considerable clinical value. Nevertheless, further research is necessary to evaluate the anesthetic techniques and their potential impact on patient outcomes.
